# Supplementary material for: Generalized Bézier-like model and its applications to curve and surface modeling
Source: PLoS One. 2024 Jun 3;19(6):e0303340. doi: 10.1371/journal.pone.0303340 (PMC11146746; doi:10.1371/journal.pone.0303340)
Supplement: Attachment — Submitted filename: Comment for PONE-D-23-44182.pdf [file pone.0303340.s001.pdf]

Manuscript ID: PONE-D-23-44182

The manuscript entitled "Generalized Bézier-like model and its applications to curve and surface modelling" proposes C^3 and G^3 continuity for curves, and G^1 and G^2 continuity for surface. This study is an extension of [1] and [2]. Hence, this work is the followed-up work. Overall, the paper has interesting results and significant contribution. However, the current of this work cannot be published until the following problems and remarks are addressed.

Minor comment:

1. There is typo in Equation (2.2) and (2.4) it should be U(Φ) since the author denoted the symbol for parametric curve.
2. Please mention that u and v are the x and y components of U(Φ), respectively for Equation (2.3) and (2.5).
3. In Equation (2.10), there is typo, it should be 0 ≤w, w1 ≤1.
4. Make sure the partition of each section is well organized. For example, Section 2.6 is for continuity of curve and surfaces but only discuss for curve only while Section 3 discuss for surface continuity.
5. In Section 3.2 first sentence, it should be G^0 continuity not G^1.
6. In Section 3.2 last sentence, it should G^2 continuity in w direction not t.
7. On page 13, last paragraph, please recheck which said figures.

Please check the manuscript for typos, grammatical mistakes, and undefined symbols.

Major remark/comment:

1. The abstract should answer the following: What is the motivation of this study? What is the current limitation of the existing method? What is the advantage of the proposed method compared to the existing method?
2. Lack of literature review regarding G^1/G^2 continuity of Bezier-like surface. Consider the following work:
   a) Hu, G., Cao, H., Wang, X., & Qin, X. (2017). G^2 continuity conditions for generalized Bézier-like surfaces with multiple shape parameters. Journal of Inequalities and Applications, 2017, 1-17.
   b) Syed Ahmad Aidil Adha Said Mad Zain, Md Yushalify Misro. A novel technique on flexibility and adjustability of generalized fractional Bézier surface patch[J]. AIMS Mathematics, 2023, 8(1): 550-589. doi: 10.3934/math.2023026
3. Introduction has unclear motivation. What is the research gap? Next, what is the novelty of this research?
4. In Section 2.4 and 2.5, there is a lack of discussion regarding the geometric effect of shape parameters to the curves and surfaces. Please give details regarding the geometric effect of shape parameters, for example what will happen if the first shape parameter is increased while the other parameter is decreased and vice versa. What would happen if both increased/decreased?
5. In Section 2.6 paragraph 1, please elaborate regarding the reason C^3/G^3 continuity is smoother than C^2/G^2. What is the significant advantage of C^3/G^3 continuity compared to C^2/G^2 in terms of visualization? Please include the curvature comb or at least curvature plot to distinguish between C^2/G^2 continuity and C^3/G^3 continuity. You may refer to these references for curvature comb:
   a) Farin, G. (2016). Curvature combs and curvature plots. Computer-Aided Design, 80, 6-8.

b) S.A.A.A. Said Mad Zain, M.Y. Misro, K.T. Miura, Curve fitting using generalized fractional Bézier curve, Computer-Aided Des. Appl. 20 (2023) 350–363.

6. In Section 2.6, it is preferable to include figures of C^2/G^2 continuity of gB-curves for comparison between C^2/G^2 and C^3/G^3 continuity.
7. In Section 3.2, 3.3 and 3.4 it is better to write the conditions and equations of G^2 continuity of surface in a proper theorem. It is quite weird because for Section 2.6 there is proper theorem for curve continuity. You can refer to reference in point (2).
8. Please recheck Eqn (3.9) and (3.10).
9. Please change title section 3.5 since it is confusing with section 3.3. Rewrite as the procedure and examples of constructing G2 surfaces using theorems.
10. Rewrite the conclusion by considering the motivation and advantages of the proposed method. Please also include the limitations of the proposed method.

If the author can address the comment, then I will recommend this manuscript for publication.
