# Supplementary material for: Generalized Bézier-like model and its applications to curve and surface modeling
Source: PLoS One. 2024 Jun 3;19(6):e0303340. doi: 10.1371/journal.pone.0303340 (PMC11146746; doi:10.1371/journal.pone.0303340)
Supplement: Attachment — Submitted filename: Reply to Comments of Reviewers.pdf [file pone.0303340.s002.pdf]

Reply to Reviewer's Comments/Suggestions

We appreciate the reviewers for the review of our manuscript. Also, we are thankful to the editors for giving us the chance to revise our manuscript. Our research is mainly " Generalized Bézier-like model and its applications to curve and surface modeling". With effort, we have revised the manuscript according to the advices from reviewers. The changes in the manuscript are in red font. Detail replies to the reviewers are listed as follows. The reviewers' opinions are in *italic* font. And our replies are in normal font for easy tracking and cross reference.

Reviewer # 01

The manuscript entitled "Generalized Bézier-like model and its applications to curve and surface modelling" proposes C^3 and G^3 continuity for curves, and G^1 and G^2 continuity for surface. This study is an extension of [1] and [2]. Hence, this work is the followed-up work. Overall, the paper has interesting results and significant contribution. However, the current of this work cannot be published until the following problems and remarks are addressed.

Minor comments:

1. *There is typo in Equation (2.2) and (2.4) it should be U(F) since the author denoted the symbol for parametric curve?*

   Answer: Thanks for your attention. The U(F) has been updated as suggested. Please see the revised manuscript.

2. *Why Please mention that u and v are the x and y components of U(F), respectively for Equation (2.3) and (2.5).*

   Answer: Done, the sentence has been updated as suggested. Please see the revised manuscript. Thank you for valuable suggestions.

3. *In Equation (2.10), there is typo, it should be 0 ≤w, w1 ≤1.*

   Answer: Modified. We have changed it as 0 ≤w, w1 ≤1. Please see the revised manuscript.

4. *Make sure the partition of each section is well organized. For example, Section 2.6 is for continuity of curve and surfaces but only discuss for curve only while Section 3 discuss for surface continuity*.

Answer: Dear professor, thank you for your comments. Your suggestion is very good for understanding the manuscript. We modified the whole manuscript as you mentioned. Please see the revised manuscript.

5. *In Section 3.2 first sentence, it should be G^0 continuity not G^1.*

Answer: Done, the sentence has been updated as suggested. Please see the revised manuscript. Thank you for valuable suggestions.

6. *In Section 3.2 last sentence, it should G^2 continuity in w direction not t.*

Answer: Revised as suggested. Please see the revised manuscript. Thank you for valuable suggestions.

7. *On page 13, last paragraph, please recheck which said figures.*

Answer: Dear professor, thank you for your comments. There is a typo mistake and we have changed it according to the figures. Please see the revised manuscript.

We have rechecked the whole manuscript and correct the all typo mistakes and grammatical errors in the manuscript.

Major remarks/comments:

1. *The abstract should answer the following: What is the motivation of this study? What is the current limitation of the existing method? What is the advantage of the proposed method compared to the existing method?*

Answer: Dear professor, thank you for your comments, we have added the motivation of our work, advantages and limitation of existing method in the abstract. Please see the revised manuscript.

The motivation of our work, limitation of existing method and some advantages over the existing methods are addressed here as:

The subject matter of surfaces in computer aided geometric design (CAGD) is the depiction and design of surfaces in computer graphics arena. Due to their geometric features, modeling of Bezier curves and surfaces with their shape parameters is the most well-liked topic of research in CAGD/computer-aided manufacturing (CAM). The primary challenges in industries such as automotive, shipbuilding, and aerospace are the design of complex surfaces. In order to address this issue, the continuity constraints between surfaces are utilized to generate complex surfaces.

The Bezier method is mostly used in the CAGD to design curves and surfaces. But there are some limitations of the Bezier method: to control the shape of the curve the points are very

difficult to adjust and if the points are changed then the shape of whole curve is disturbed. There is another issue that it is very difficult to design complex curves and difficult to handle the curve of our choice.

To address this issue the new basis function with two different shape parameters is introduced. By changing the value of shape parameters, the shape of the curve is controlled. To design the complex curves in different industries the continuity constraints between two generalized Bezier-like surfaces with different shape parameters are proposed which is very useful in modeling the surfaces.

2. *Lack of literature review regarding G^1/G^2 continuity of Bezier-like surface. Consider the following work:*
   a) Hu, G., Cao, H., Wang, X., & Qin, X. (2017). G^2 continuity conditions for generalized Bézier-like surfaces with multiple shape parameters. Journal of Inequalities and Applications, 2017, 1-17.
   b) Syed Ahmad Aidil Adha Said Mad Zain, Md Yushalify Misro. A novel technique on flexibility and adjustability of generalized fractional Bézier surface patch[J]. AIMS Mathematics, 2023, 8(1): 550-589. doi: 10.3934/math.2023026

Answer: Dear professor, thanks for your concerned. We have added the more literature as you suggested. The papers are added in the literature review. Please see the revised manuscript.

3. *Introduction has unclear motivation. What is the research gap? Next, what is the novelty of this research?*

Answer: The introduction has been updated as suggested. Please see the revised manuscript. Thank you for valuable suggestions.

4. *In Section 2.4 and 2.5, there is a lack of discussion regarding the geometric effect of shape parameters to the curves and surfaces. Please give details regarding the geometric effect of shape parameters, for example what will happen if the first shape parameter is increased while the other parameter is decreased and vice versa. What would happen if both increased/decreased?*

   Answer: Dear professor, thank you for your valuable comments. The effect of shape parameters on the curves and surfaces is discussed, and to check the effect of shape parameters, some curves and surfaces are also added. It can be seen that, by changing the value of shape parameters, the curves and surfaces are changed and can be altered by our choice. As the value of shape parameters increases, the curves and surfaces get closer to the control net.

5. *In Section 2.6 paragraph 1, please elaborate regarding the reason C^3/G^3 continuity is smoother than C^2/G^2. What is the significant advantage of C^3/G^3 continuity*

compared to C^2/G^2 in terms of visualization? Please include the curvature comb or at least curvature plot to distinguish between C^2/G^2 continuity and C^3/G^3 continuity. You may refer to these references for curvature comb:
a) Farin, G. (2016). Curvature combs and curvature plots. Computer-Aided Design, 80, 6-8.
b) S.A.A.A. Said Mad Zain, M.Y. Misro, K.T. Miura, Curve fitting using generalized fractional Bézier curve, Computer-Aided Des. Appl. 20 (2023) 350–363.

Answer: Modified as suggested. Please see the revised manuscript**.** Thank you for valuable suggestions.

6. *In Section 2.6, it is preferable to include figures of C^2/G^2 continuity of gB-curves for comparison between C^2/G^2 and C^3/G^3 continuity*.

Answer: Dear professor, thank you for your valuable comments. For the comparison of C^2/G^2 and C^3/G^3 continuity of gB-curves, figures are added. Please see the revised manuscript.

7. *In Section 3.2, 3.3 and 3.4 it is better to write the conditions and equations of G^2 continuity of surface in a proper theorem. It is quite weird because for Section 2.6 there is proper theorem for curve continuity. You can refer to reference in point (2).*

Answer: Dear professor, thank you very much for your comments, the sections have been updated as suggested. Please see the revised manuscript.

8. *Please recheck Eqn (3.9) and (3.10).*

Answer: Dear professor, thank you very much for your comments. There is a typo mistake and the equations has been changed. Please see the revised manuscript.

9. *Please change title section 3.5 since it is confusing with section 3.3. Rewrite as the procedure and examples of constructing G2 surfaces using theorems.*

Answer: Changed. Thank you for your valuable suggestion. Please see the revised manuscript

10. *Rewrite the conclusion by considering the motivation and advantages of the proposed method. Please also include the limitations of the proposed method.*

Answer: The conclusion section has been updated according to the motivation. The advantages of the proposed method were also discussed. In addition, there are some limitations, and future work also added. Please see the revised manuscript.

Thank you very much for your valuable comments and suggestions

Reviewer # 02

This paper is about the explanation of a novel Bezier-like curve definition. Apart from a few grammar errors, the text is well-written. However, it must be checked (again) by a native English-speaker. They used Bernstein-like functions as the basis of their construction. I didn't check all the equations and calculations of this paper. It looks like doable anyways. Therefore, I am more concerned with the idea and the novelty of the method this study proposes. I find them original and see it as positive contributions to the field.

Answer: We have rechecked the whole manuscript and the corrections have been done. Please see the revised manuscript**.** Thank you for your valuable suggestions.

Thank you very much for your valuable comments and suggestions

Reviewer # 03

This article extends the previous work of Ameer et al. (2022a, 2022b) by introducing Generalized Bezier-like C^3 and G^3 curve and surface design. Overall acceptable, but there are many typos exist in the present version. Below are comments to improve the paper.

Thank you very much for your recommendation

1. *Abstract: there is no evident in the article to support the proposed method is better than the standard Bézier curves and surfaces. This is the major issue in this article, show clearly how the proposed curve/surface better than traditional Bézier model.*

   Answer: Dear Professor, thank you for your comments. The abstract is changed as suggested. Here is a note on the advantages of the proposed method over standard Bézier model:
   The standard Bézier curves and surfaces have no shape parameters, while our proposed method has two different shape parameters, which helps us alter the shape of our curves and surfaces according to our choice. In the standard Bézier model, the adjustment of the curves and surfaces is very difficult and also difficult to control. In the proposed method, the control of the curves and surfaces is easy due to the addition of shape parameters. Please see the revised manuscript.

2. *pg.2: Before writing the short form, make sure to write the full form, e.g "H-BS", "GHT-BS" etc*

   Answer: The sentence has been updated as suggested. Please see the revised manuscript.

3. *pg.2: the contribution of this paper is the continuity extension, the rest of the points are either redundant or has been established in Amber et al. (2022a, 2022b).*

   Answer: Dear Professor, thank you for your comments. The sentence has been updated as suggested. Please see the revised manuscript.

4. *Eq. (2.1) should be written as M =(u,v); this notation is in line with the rest of equations.*

   Answer: Modified as suggested. Please see the revised manuscript. Thank you for valuable suggestions.

5. *pg.3: U(Φ) should be written as M(Φ).*

   Answer: Dear Professor, thank you for your comments. There is a typo mistake and it is corrected in the whole manuscript. Please see the revised manuscript.

6. *what is gB-like? state the full form first.*

Answer: Stated as suggested. Please see the revised manuscript. Thank you for valuable suggestions.

7. *pg.4: define C_f stated in eq. (2.8).*

   Answer: Dear Professor, thank you very much for your comments. We have defined the C_f as the worthy reviewer suggested. Please see the revised manuscript.

8. *eq.(2.10) typo: "0<=w,w1<=1", not "0<=w,w1<=r"*

   Answer: Dear Professor, thank you for your concern. We have changed it as suggested. Please see the revised manuscript.

9. *replace "talk" with "discuss".*

   Answer: Modified as suggested. Please see the revised manuscript. Thank you for your valuable suggestions.

10. *Theorem 1: state for "parametric continuity C^3", the rest has been established in Amber et al. (2022a, 2022b).*

    Answer: According to the suggestion of worthy Professor, the sentence has been updated. Please see the revised manuscript.

11. *C^f or C_f? be consistent.*

    Answer: Dear Professor, thank you for your concern. We have changed it throughout the manuscript and used just a single template. Please see the revised manuscript.

12. *Theorem 2: state for "parametric continuity G^3", the rest has been established in Amber et al. (2022a, 2022b).*
    Answer: According to the suggestion of worthy Professor, the sentence has been updated. Please see the revised manuscript.

13. *what is φ?*

    Answer: Dear Professor, there is a typo mistake and we have changed it. Please see the revised manuscript.

14. *Typo: the Fig.1, not Fig.2.*

    Answer: Dear Professor, thank you for your concern. The sentence has been updated as suggested by a worthy Professor. Please see the revised manuscript.

15. *State all the details to produce Figure 1. This applies for the rest of figures too, hence increasing reproducibility.*

    Answer: Dear Professor, this figure is constructed with the help of continuity constraints in C3. The control points of the first curve are given by the user, and the first four control points are calculated by the continuity constraints of C3, and the remaining points of the second curve are given by the user. The details of all figures are added to the manuscript. Please see the revised manuscript.

16. *Equations stated after eq. (3.10) has typo: replace d(z) with d(w).*

    Answer: Dear Professor, there is a typo mistake, and we have changed it according to the variable used for the scaling factor. Please see the revised manuscript.

17. *typo in eq. (3.12), m1?*

    Answer: Modified. Thank you for your valuable suggestions. Please see the revised manuscript.

18. *typo in equation citing: Substituting (3.12) and (3.13) into (3.25)?*

    Answer: Respected Professor, yes, it is typo mistake and the mistake has been corrected as you suggested. Please see the revised manuscript.

19. *pg.13: typo Fig.3(a)....Fig.3(b)*

    Answer: Respected Professor, it is typo mistake and the mistake has been corrected as you suggested. Please see the revised manuscript.

Thank you very much for your valuable comments and suggestions
